# Relationship between Gastric Ulcer and Cancer

**Published:** 1917-04

**Authors:** 


**Relationship Between Gastric Ulcer and Cancer.** Frank Smithies, Chicago, Ill. Med. Jour., March. In 1882, Zenker expressed the opinion that all gastric cancers arose on the basis of pre-existing benign ulcers. In 1889 Rosenheim concurred. In 1902, Futterer held that cancers frequently arose on ulcers of the pylorus but less commonly in other regions. Other observers have made the following estimates: Fenwick 3%, Friedenwald 7.3%, Moynihan 66%, Sapaska 10%, of cancer cases traced back to ulcers. Smithies, in 953 cases of gastric cancer, demonstrated by operation or necropsy, found that 543 (57%) gave histories of ulcer and 93 more (9%) of atypic ulcer. 544 cases of benign ulcer had an average duration of 11 years. 646 ultimately malignant cases had an average duration of dyspeptic period of ulcer type of 10.8 years. 93 cases of cancer following atypic ulcer symptoms gave an average duration of the clinically malignant period of 6 months. 337 cases, clinically malignant from inception, had an average duration of 6.9 months.

